# Advances in adhesion interfaces of peripheral nerve repair materials: mechanism, classification, evaluation method, and functionalization

**DOI:** 10.3389/fcell.2025.1725351

**Published:** 2025-12-12

**Authors:** Qi Meng, Meng Zhang, Yichong Zhang, Haoran Jiang, Yang Qu, Ci Li, Chenyujun Hu, Zhihao Lin, Bohan Xing, Fengxue Zhu, Peixun Zhang

**Affiliations:** 1 Department of Orthopedics and Trauma, Peking University People’s Hospital, Beijing, China; 2 National Centre for Trauma Medicine, Beijing, China; 3 Key Laboratory of Trauma and Neural Regeneration, Peking University, Beijing, China; 4 Beijing Laboratory of Trauma and Nerve Regeneration, Peking University, Beijing, China; 5 Department of Orthopedics, Shandong Provincial Hospital Affiliated to Shandong First Medical University, Jinan, Shandong, China; 6 Department of Intensive Care Medicine, Trauma Center, Peking University People’s Hospital, Beijing, China; 7 Department of Trauma and Orthopedics, Peking University People’s Hospital Qingdao Hospital, Qingdao, Shandong, China

**Keywords:** peripheral nerve injury, nerve repair material, adhesion interface, wet adhesion, functionalization

## Abstract

Peripheral nerve injury is a prevalent clinical disease, but achieving functional recovery remains a significant research challenge. In recent years, the therapeutic efficacy of nerve repair materials has garnered widespread attention from researchers. Notably, the adhesion interface between the repair material and the nerve tissue stands as a critical factor affecting the outcome of nerve regeneration. In this review, we firstly outline the importance of adhesion interface in nerve repair; subsequently, we systematically summarize the typical adhesion mechanisms; in addition, we discuss the design of adhesion interfaces for natural and synthetic materials, as well as the dry and wet adhesion strategies. To address the *in vivo* wet environment, emphasis is placed on the adhesion design of wet-adhesive materials and related research progress. Furthermore, methods to evaluate adhesion property and strategies for materials functionalization are also provided. Through summarizing the current research findings, this paper aims to provide theoretical basis and technical reference for the optimal de-sign of interfaces of nerve repair materials, thereby facilitating the clinical translational application of peripheral nerve injury repair materials.

## 1 Introduction

Peripheral nerve injury (PNI) is a common disease in which the peripheral nerve plexus, nerve trunks, and their branches are structurally damaged, with the most common cases occurring after trauma or surgical operations (Chu et al., 2022). Although the nervous system is capable of recovering after injury, the ability is limited and the speed of recovery is relatively slow (Previtali, 2021). When the nerve injury defects >3 cm, autografts and allografts would be recognized as “gold standard” treatment option. Unfortunately, autologous grafts have limited donors and carry risks such as neuroma formation, while allogeneic grafts may result in immune rejection and disease transmission. In recent decades, significant progress has been made in the research and application of tissue-engineered materials in the field of nerve injury repair (Ma et al., 2024; Mankavi et al., 2023).

Adhesion interface is one of the key factors determining whether a repair material can effectively perform its biological function (Kong et al., 2022). The therapeutic potential of nerve repair materials depends largely on the formation of a stable and long-lasting connection at the tissue-biomaterial interface (Joshi et al., 2023). Excellent attachment at the interface not only avoids instability over time, but also effectively withstands the stresses generated by the movement of the nerve tissue, thus achieving long-term stable fixation and long-lasting performance of the graft (Jin et al., 2025). In addition, favorable adhesives can tightly bridge the nerve severed ends to constitute a dense space, which could simplify the complicated and delicate suturing procedures, and also contribute to shorter surgical time and the minimization of local trauma and inflammatory reactions. Therefore, the field of adhesion interfaces has received growing research interest in recent years, and a variety of novel adhesives have been successfully developed for different application situations of nerve injury repair.

In this paper, we reviewed advances in the researches of adhesion interfaces for tissue engineering materials in PNI repair. First, the significance of adhesion in nerve repair was outlined; subsequently, we introduced the adhesion mechanisms involved; next, we separately discussed the adhesion interfaces of natural and synthetic materials, as well as dry adhesion and wet adhesion. Furthermore, current methods for evaluating the adhesion properties were presented, and functionalization strategies for imparting biological features to materials were provided. Finally, potential future directions for adhesion interface research in this field were proposed, aiming to offer valuable references for enhancing the clinical and therapeutic efficacy of nerve repair.

## 2 Significance of adhesion interface

Robust adhesion ensures a good connection between two interfaces and prevents these substrates from dissociating. Tissue adhesives have been studied and applied as early as the 1950s (e.g., for simple wound closure) and have received increasing attention over the past decades (Bal-Ozturk et al., 2021). Currently, micro-suture techniques are still commonly used in the repair of peripheral nerve severance, both in simple suture and nerve grafting protocols. By contrast, the use of adhesive nerve repair materials can significantly reduce the reliance on traditional suture techniques and simplify the surgical process by reducing the need for expensive suture instruments and sophisticated microsurgical techniques (Qiu et al., 2024). Meanwhile, because of the fragility of the nerve tissue, the suture operation inevitably causes mechanical damage, which in turn triggers inflammatory reactions, fibrous tissue proliferation and scar formation, hindering myelin regeneration, and ultimately may lead to nerve repair failure (Isaacs et al., 2009). Adhesion interfaces provide a feasible solution to this problem. Previous studies have demonstrated that nerve repair adhesives can significantly lower the levels of inflammatory mediators (e.g., TNF-α and IL-6) and inhibit fibrosis, thereby fostering a favorable microenvironment for nerve regeneration (DiStefano et al., 2020; Jha et al., 2023). Furthermore, the strong adhesion secures the repair material at the injury site, thereby enabling the precise local delivery of drugs and bioactive molecules (Qiu et al., 2024). By modulating the physical and chemical properties of the adhesive materials, the degradation rate can be matched with the nerve repair period to achieve the sustained release of drugs (Gnavi et al., 2017), and even personalized functional customization can be accomplished according to the type of injury and expected efficacy.

## 3 Mechanism of adhesion interface

The interfacial connection is the fundamental factor of the adhesion process, and investigating its underlying mechanisms is crucial for achieving the clinical translation of nerve repair materials (Bal-Ozturk et al., 2021). The primary mechanisms of adhesion include mechanical interlocking, electrostatic binding, intermolecular bonding, and diffusion. The successful realization of adhesion is usually the combination of multiple mechanisms (Zhu et al., 2018).

### 3.1 Mechanical interlocking

**FIGURE 1:**
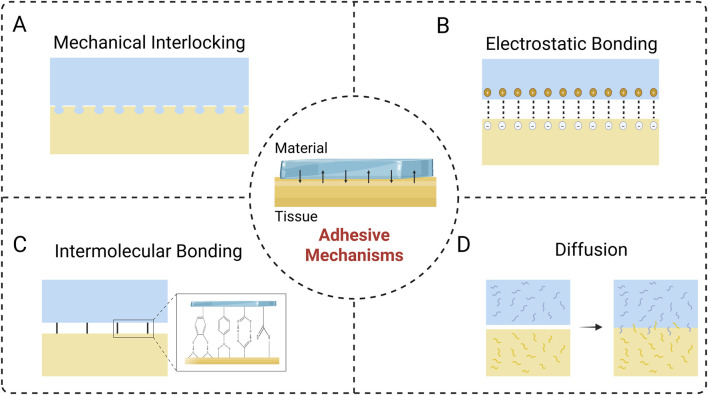
Adhesive Mechanisms at the material-tissue interface. **(A)** Mechanical interlocking. **(B)** Electrostatic bonding. **(C)** Intermolecular bonding. **(D)** Diffusion.

In fact, mechanical interlocking has already been studied in adhesion theories (Predecki et al., 1972). It is essentially a biophysical phenomenon at the interface between the adherent material and the tissue, manifested as the penetration of the material into the pores and irregularities of the surface of adherent to achieve adhesion (Figure 1A). Mechanical interlocking can be visualized by means of an optical microscope or an electron microscope (DiStefano et al., 2020). As biomaterials and tissue surfaces contact each other, at the microscopic scale, the air accommodated by the roughness of the tissue surface is extruded, allowing the materials to immerse and interdigitation, and ultimately completing the interfacial connection (Bal-Ozturk et al., 2021; DiStefano et al., 2020). Therefore, offering optimal interfacial morphology characteristics via surface modification is crucial, including roughness, contact area, and porosity (Bal-Ozturk et al., 2021). For instance, Jang et al. used microscale and nanoscale techniques to empower titanium-based dental implants with multiscale roughness, thus significantly improving the mechanical interlocking effect between the material-tissue interface and allowing for more stable adhesion and fixation (Jang et al., 2017). Also, inspired by internal parasites, Yang et al. designed and prepared a microneedle consisting of a swellable tip and a non-swellable core, which can realize adhesion of the material through a mechanical interlocking. This microneedle is minimally traumatic and has a low risk of infection. It enables secure fixation to various tissue surfaces, such as skin and intestinal mucosa, and facilitates effective drug delivery, demonstrating significant application potential (Yang et al., 2013).

### 3.2 Electrostatic bonding

Electrostatic bonding arises from the molecular scale attraction between opposing charges on the material and biological tissue surfaces, which forms a robust adherence (Figure 1B) (Bal-Ozturk et al., 2021). The strength of the adhesion relies on the surface charge density, which can be managed by adjusting the ionic content of the medium surrounding the material. However, in the presence of an insulator component, the contribution of electrostatic binding to adhesion is weak due to the slow process of charge accumulation and the scarcity of available electrons (Pinnaratip et al., 2019). In contrast, this adhesion mechanism is applicable to materials based on polymers or metals (Bal-Ozturk et al., 2021). Tian and colleagues utilized 2-acrylamido-2-methylpropane sulfonic acid to enhance electrostatic interactions, thus constructing a hydrogel that could firmly adhere to the surface of rat muscle and heart, and enabling the recording of electrophysiological signals (Tian et al., 2022). In the aqueous environment, inorganic solid surfaces (e.g., rocks, metals) and biological systems (cell membranes, tissues) are commonly negatively charged (Fan et al., 2019; van den et al., 2011), thus electrostatic interactions can serve as one of the key mechanisms for achieving wet adhesion. The interaction of aromatic amino acids with positively charged regions allows them to attach and aggregate on anionic surfaces, which is related to cation-π interactions (Vanhoye et al., 2004). Mussel foot proteins (MFPs) is an ideal reference model for the development of wet adhesives due to its prominent cation-π interaction ability. Cai et al. prepared a wet-adhesive hydrogel, which can exert strong wet adhesion efficacy through electrostatic interactions and hydrogen bonding between interfaces, being able to effectively adhere to nerve tissue and promote nerve repair (Cai C et al., 2022).

### 3.3 Intermolecular bonding

Intermolecular bonding is generated by intermolecular forces at the interface between the adhesive material and the surface of the biological tissue (Figure 1C). Biological tissues contain various functional moieties such as carboxyl, sulfur, amino, and hydroxyl groups, which can serve as anchoring sites to form strong connections with materials through chemical forces (e.g., ionic, covalent, and metallic bonds) and physical forces (e.g., van der Waals forces) (Bal-Ozturk et al., 2021; DiStefano et al., 2020). Adhesion mediated by chemical forces is generally stronger than that achieved through physical interactions, and the covalent bonding is the most common type in chemical force adhesion mechanism, such as imine bond, amide bond, disulfide bond (Lee et al., 2023). With surface modification or bulk modification, it is feasible to introduce specific chemical functional groups into the chemical structure of the material, thereby facilitating the covalent binding of the material to biological tissues and enhancing the adhesion efficacy (Lee et al., 2023; Lei et al., 2023). Physical forces are derived from non-covalent intermolecular interactions, in which van der Waals forces might play a significant role (Ngo and Grunlan, 2017). Physical adsorption strength is closely related to the physical properties of the material surface, and abundant bonding sites would provide favorable adhesion (Bal-Ozturk et al., 2021; Mehdizadeh and Yang, 2013). Wang et al. developed an adhesive nerve hydrogel bandage fabricated from extracellular matrix, oxidized polysaccharides (OS), poly(3,4-ethylenedioxythiophene)/ poly(styrenesulfonate) (PEDOT:PSS), and indole-3-propionic acid (IPA). The firm adhesive property of the bandage resulted from the covalent reaction between the aldehyde group in OS and the amino group on the surface of the tissue, combined with the hydrogen bonding effect, allowing the bandage to tightly adhere to the nerve fibers without the need for sutures, and ultimately regenerating axons and restoring sensory functions by modulating neutrophil chemotaxis and electrical signaling (Wang et al., 2023).

### 3.4 Diffusion

Diffusion occurs at the material-tissue interface, where the molecules of the adhesive material and the biological tissue interdiffuse, thus forming an integrated layer (Figure 1D) (Bal-Ozturk et al., 2021; DiStefano et al., 2020). Therefore, favorable diffusive adhesion requires excellent material biocompatibility and sufficient mobility of the polymer chains (Bal-Ozturk et al., 2021; von Fraunhofer, 2012). In addition, diffusive adhesion relies on the interaction of molecules on the surface, so sufficient contact time ensuring an enough diffusion to take place is also essential for adhesion (von Fraunhofer, 2012). Su et al. fabricated an injectable hydrogel with urea as one of the components, which achieved efficient, strong and long-lasting adhesion performance with diffusion of urea molecules (Su et al., 2021).

## 4 Classifications of adhesion interface

The design and selection of adhesion interface are pivotal factors determining the efficacy of peripheral nerve repair. Based on material properties, adhesion interface can be broadly categorized into two types: natural polymer-based and synthetic polymer-based. Depending on the adhesion environment, adhesion interface can further be classified as dry or wet adhesion interface. Dry adhesion interface primarily function in dry or low-humidity environments; wet adhesion interface, however, require overcoming interference from tissue surface fluids, making them a key research focus and challenge for achieving efficient, stable *in vivo* adhesion.

### 4.1 Natural and synthetic polymer-based adhesion interface

#### 4.1.1 Natural polymer-based adhesion interface

**FIGURE 2:**
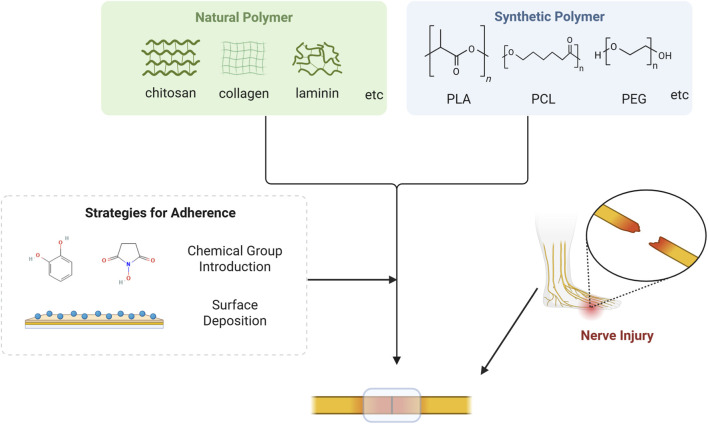
Mechanism for natural or synthetic polymer to repair nerve injury. Natural polymer based on chitosan, collagen, laminin and etc., as well as synthetic polymer based on PLA, PCL, PEG and etc., can acquire robust adhesion capacity via strategies such as chemical group introduction and surface deposition. Consequently, these materials can adhere to the surface of the damaged nerve and facilitate nerve regeneration.

Materials based on natural polymers have developed significantly over the past decades, with numerous reported applications in the field of repairing damaged nerve, muscle, bone, and other tissue (Bu et al., 2023; Yuan et al., 2023). Natural polymer adhesives (especially fibrin glue) have been studied for nerve impair for a long time, including chitosan, collagen, laminin, etc (Figure 2) (Correia et al., 2023). Biomaterials constructed from natural polymers usually possess excellent biocompatibility and outstanding biological activity, but are often deficient in the aspect of mechanical or functional properties (Amalakanti et al., 2024). In clinical applications, there are often concerns about their insufficient adhesive capacity when used alone, which in turn leads to diminished therapeutic efficacy (Temple et al., 2004). It has become an important and feasible way to guarantee their functionality by modifying their surfaces with a diverse range of chemical groups. At the material-nerve interface, the rich amine, hydroxyl, carboxyl, or modified reactive groups, such as N-hydroxysuccinimide (NHS) esters and catechol group, in the material can bond with the chemical groups on the tissue surface (Bal-Ozturk et al., 2021; Bhagat and Becker, 2017). To repair short-gap nerve defects, Dong et al. prepared a rapidly-formable chitosan hydrogel, which achieved robust adhesion by grafting catechol groups on the surface of the hydrogel (Dong et al., 2024). Zhou et al. constructed an in situ-formed neural adhesion hydrogel using chitosan and ε-polylysine, conferring strong adhesion properties to the material via linking catechol groups to the polylysine backbone, thus realizing excellent neural repair effects (Zhou et al., 2016). Xue and colleagues developed a dual-network neural adhesive, the first network formed by cross-linking dopamine-isothiocyanate-modified hyaluronic acid via thiourea-quinone coupling reaction, and the second constructed by self-assembly of decellularized nerve matrix. The adhesive attach to the nerve endings through covalent bonding of catechol to amines or thiols on the nerve surface or noncovalently hydrogen bonding, thus providing robust adhesion (Xue et al., 2023).

#### 4.1.2 Synthetic polymer-based adhesion interface

Synthetic polymer-based materials include polylactic acid (PLA), polycaprolactone (PCL), polyethylene glycol (PEG), etc (Amalakanti et al., 2024) (Figure 2). Compared to natural polymers, synthetic polymers usually have superior mechanical strength, controlled degradation rates, and favorable modifiability, allowing them to better simulate the physical and chemical properties of neural tissue (Table 1). However, synthetic polymer-based materials tend to suffer from higher biotoxicity (Amalakanti et al., 2024; Bal-Ozturk et al., 2021). PLA has high mechanical resilience and excellent molding and shaping properties, thus it is commonly used in the preparation of nerve regeneration conduits (Liu et al., 2017). Nevertheless, PLA is strongly hydrophobic and its degradation by-product, lactic acid, may trigger a localized inflammatory response (Bao et al., 2024). A study reported that PLA-based conduit facilitated the repair of injured sciatic nerve, representing a promising alternative to nerve suturing (Goulart et al., 2016). PCL is an aliphatic polyester with good biocompatibility and a relatively long degradation cycle of up to 24 months (Vijayavenkataraman et al., 2019). Due to its superior melt viscosity and low melting point, PCL is ideally suitable for the preparation of polymers through 3D printing (Bruyas et al., 2018). Taylor et al. employed a surface deposition method to modify PCL fiber scaffolds with oxygen plasma and 11-aminoundecyltriethoxysilane, thereby enhancing surface roughness and adhesion, which ultimately improved nerve repair (Taylor et al., 2023). PEG, a widely-used biomaterial, also possesses favorable biocompatibility and highly adjustable physical properties (Annabi et al., 2015). Despite its weak adhesion ability, adhesives with PEG as the main ingredient still attract attention. Modification of some chemical groups (e.g., NHS and amine groups) or the combination of polysaccharide or protein-based adhesives will allow for improved performance (Bal-Ozturk et al., 2021; Kelmansky et al., 2017). Amoozgar and colleagues prepared a hybrid hydrogel of 4-aminobenzoic acid-modified chitosan (Az-C) and PEG by photocross-linking. The excellent bioadhesive and *in situ* cross-linking properties of Az-C and the good mechanical properties of PEG equipped the hydrogel with powerful adhesion and high mechanical strength simultaneously, which enabled a long-lasting treatment of nerve injury (Amoozgar et al., 2012).

**TABLE 1:** Comparison between natural and synthetic polymer adhesion interface.

Category	Natural polymer-based adhesion interface	Synthetic polymer-based adhesion interface
Examples	Chitosan, collagen, laminin, etc	PLA, PCL, PEG, etc.
Advantages	Excellent biocompatibility; Outstanding biological activity	Superior mechanical strength; Controllable degradation; Tunable physical properties
Limitations	Weak mechanical properties; Rapid degradation	Higher biotoxicity; Hydrophobicity
Representative Studies	Catechol-grafted chitosan hydrogel (Dong et al., 2024); Chitosan/polylysine hydrogel (Zhou et al., 2016); Dual-network hyaluronic acid/ decellularized nerve matrix adhesive (Xue et al., 2023)	Oxygen plasma-modified PCL (Taylor, et al., 2023); Az-C/PEG hybrid hydrogel (Amoozgar et al., 2012)

### 4.2 Dry and wet adhesion interface

#### 4.2.1 Dry adhesion interface

Biological adhesion is divided into two categories: dry adhesion and wet adhesion (Borijindakul et al., 2021). In the nature, dry adhesion enables some animals to fight against the gravitational force to accomplish walking or climbing on the surface of walls and ceilings freely, such as spiders and geckos (Borijindakul et al., 2021). Such dry adhesion arises from the intermolecular adhesion formed between deformable setae on their body surfaces and the contacting surface (Tian et al., 2006), while dry adhesives have been studied and applied in a variety of fields such as robotics, medical device manufacturing and aerospace (Hassani and Baji, 2023). However, when dealing with wet biological tissues, wet adhesives tend to serve a more critical function than dry adhesives.

#### 4.2.2 Wet adhesion interface

**FIGURE 3:**
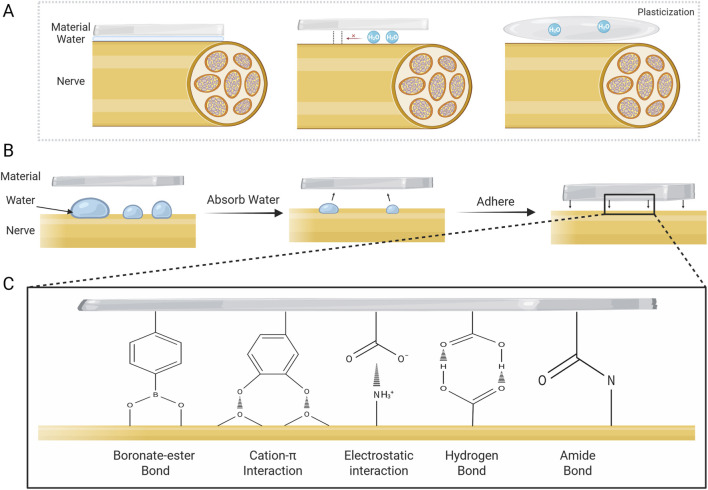
Challenges to adhesion posed by water and mechanism of wet adhesion. **(A)** Negative effects of water on adhesion. **(B)** Typical adhesion mechanism of wet-adhesion interface. Wet adhesives adsorb water on tissue surface and swell, then adhere to damaged nerve via various mechanisms. **(C)** Representative adhesion mechanisms at the material-tissue interface for wet adhesives.

During nerve injury repair, the moist tissue microenvironment poses serious challenges to the adhesive strength of materials (Figure 3A), requiring the development of biomaterials with excellent wet adhesion properties (Luo et al., 2024). The negative impact of water on the adhesion performance of materials is primarily in three ways: first, in the macro scale, the water on the surface of the tissue can significantly narrow the actual contact area between the material and the tissue (Fan and Gong, 2021; Luo et al., 2024); second, at the micro level, the hydration layer formed by the water at the material-tissue interface would weaken the molecular interactions and suppress the formation of effective chemical and physical bonds (Chen et al., 2023; Liu et al., 2023); and third, the penetration of water into the material may lead to the plasticization, or hydrolysis of the polymer network, reducing the cohesive strength and structural stability of the material, and ultimately leading to the failure of long-lasting adhesion (Chen et al., 2023; Luo et al., 2024).

Recent years have seen major advances in hydrogel materials with interfacial water-absorbing capabilities (Lee et al., 2024). These materials can efficiently remove interfacial water, thereby eliminating the hydrational layer while exhibiting excellent tissue compatibility. They achieve robust adhesion to nerve tissue surface through mechanisms including electrostatic interactions and covalent bonding (Table 2) (Figures 3B,C) (Lee et al., 2024). Jin et al. reported an adhesive conductive nerve bandage containing hyaluronic acid hydrogel fibers conjugated with phenylborate. In the aqueous environment, the dry bandage spontaneously swells into a wet adhesive upon contact with the tissue, and subsequently tightly wraps the nerve tissue by means of the self-healing property of the dynamic boronate-ester bond, which enables a firm adhesion without the need for suturing (Jin et al., 2025). Seong and colleagues proposed a sticky, strain-gradient artificial epineurium consisting of a viscous hydrogel layer and two hydrophobic elastic layers. When combining dissected nerves, its low-modulus dehydrated layer (alginate coupled with boric acid) rapidly swells by absorbing water. This process activates abundant hydrogen bonds between the alginate skeleton and epineurium, and simultaneously triggers gelation via dynamic boric acid-diol cross-linking, forming a strongly wet-adhesive hydrogel. This system presents a promising alternative to conventional micro-suture technique (Seong et al., 2024).

**TABLE 2:** Wet-adhesion biomaterials for nerve injury repair.

Material	Key component	Wet adhesion mechanism	References
Nerve bandage	Phenylborate-conjugated hyaluronic acid hydrogel	Spontaneously swells into a wet adhesive, and wraps the nerve tissue via dynamic boronate-ester bond	Jin et al. (2025)
Artificial epineurium	Viscous hydrogel layer (alginate coupled with boric acid)	Alginate-boric acid layer absorbs water, activates hydrogen bonds with epineurium; dynamic reversible cross-linking under physiological pH	Seong et al. (2024)
Nerve guidance conduits	Poly(cationic-π)/catechol-based wet-adhesive hydrogel	Electrostatic interactions and hydrogen bonds between interfaces	Cai C et al. (2022)
Bioinspired hydrogel	Hyaluronic acid-graft-dopamine (HADA)	Catechol groups in HADA form covalent bonds with nucleophilic groups	Tan et al. (2024)
Dual-layer adhesive patch	Bioadhesive layer based on Silk fibroin, tannic acid, and polyethylene glycol	Catechol groups in bioadhesive layer form strong bonds with nucleophilic groups	Zheng et al. (2025)
Conductive hydrogel	Polyacrylamide–poly(acrylic acid) (PAM–PAA) and polydopamine-modified carbon nanotubes	PAA absorbs water and forms hydrogen bonds/electrostatic interactions; Catechol groups and NHS esters in PDA form covalent bonds with tissue groups	Chu et al. (2025)

In addition, the biomimetic materials based on the natural wet adhesion mechanism have been hot research topics in the field of biomedical materials (Balkenende et al., 2019; Stark and Mitchell, 2019). Notably, biomaterials developed based on mussel biomimetic mechanisms hold great promise for application in disease treatment and tissue repair (Li K et al., 2021). Mussels’ wet adhesion is based on MFPs, which are rich in catechol groups and cationic amino acids (such as lysine) that can form covalent bonds with nucleophilic groups and generate cation-π interactions, enabling excellent adhesion in moist physiological environments (Bal-Ozturk et al., 2021; Chen et al., 2023). Inspired by the natural phenomenon, researchers have developed various bio-inspired adhesive materials by mimicking the catechol chemistry and cation-π interaction mechanisms of mussels, demonstrating significant therapeutic potential in the field of neural tissue repair. Cai and colleagues formulated a poly(cationic-π)/catechol-based wet-adhesive hydrogel nerve guidance conduit. This material achieves strong adhesion in a wet environment via electrostatic interactions and the formation of hydrogen bonds between surfaces, which can effectively wrap and adhere to damaged nerves, and thus promote nerve regeneration and functional recovery (Cai C et al., 2022). Tan et al. prepared a biomimetic hydrogel by incorporating hyaluronic acid-graft-dopamine ((HADA) with a designer peptide HGF-(RADA)4-DGDRGDS. In the wet environment, the catechol groups in HADA could form molecular covalent bonds with nucleophilic groups such as amino and hydroxyl group on the spinal cord, realizing favorable attachment to the spinal cord (Tan et al., 2024). Similarly, Zheng and colleagues proposed a silk fibroin (SF)-based dual-layer adhesive patch consisting of a bioadhesive layer and a hydrogel matrix layer. The bioadhesive layer is composed of SF, TA, and PEG, with catechol groups forming a strong bond with nucleophilic groups on the surface of the wet tissue to achieve strong and long-term interfacial adhesion, which can effectively promote axonal growth and myelin regeneration, and thus demonstrates a good potential for clinical application (Zheng et al., 2025). In addition, a study reported a multifunctional hydrogel based on polyacrylamide-poly(acrylic acid) (PAM-PAA) substrate, enhanced by polydopamine-grafted carbon nanotubes. In this hydrogel, the PAA could absorb water at the wet tissue interface and form physical cross-links such as hydrogen bonds and electrostatic interactions with the tissue through carboxyl groups; at the same time, the catechol groups in PDA can covalently bond with the amine and thiol groups on the tissue surface, and the NHS ester group could form covalent cross-links with the primary amino groups of tissue surface via the coupling reactions. With these effects, the hydrogel can obtain stable long-term adhesion, consequently ensuring a stable bioelectronic neural interface (Chu et al., 2025).

Wet adhesion offers a promising suture-free strategy for nerve repair, but clinical translation still faces multiple challenges. The dynamic, continuous aqueous environment within the body demands materials capable of long-term resistance against water molecules’ disruption of interfacial bonding. In addition, peripheral nerves exist within complex biomechanical microenvironment, requiring materials to possess sufficient interfacial toughness, thereby preventing adhesion failure due to stress concentration. Furthermore, the long-term biocompatibility of chemical groups introduced for enhanced adhesion with neural tissue remains to be evaluated. To address these challenges, future efforts should focus on developing multi-mechanism synergistic adhesion strategies to create novel biomaterials that combine durable adhesion with mechanical toughness. Meanwhile, establishing more clinically relevant evaluation systems to assess long-term safety in physiological environments is essential to advance their translation into clinical applications.

## 5 Evaluation methods of adhesion property

**FIGURE 4:**
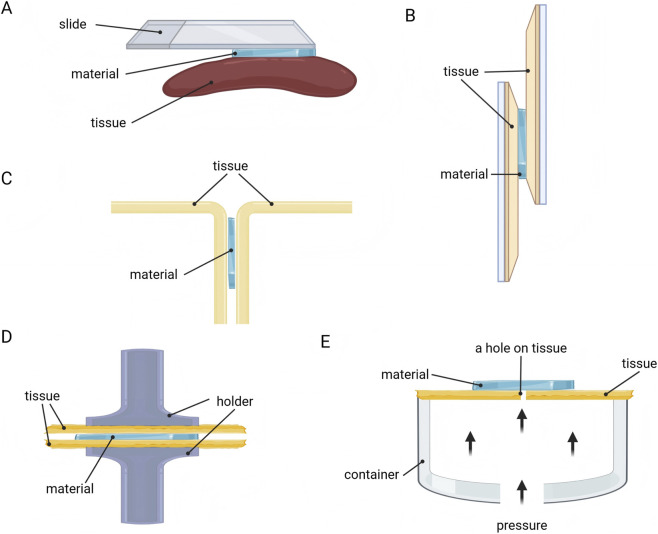
Methods for evaluating adhesion property of nerve repair materials. **(A)** Visual assessment. **(B)** Lap-shear test. **(C)** Peeling test. **(D)** Tensile test. **(E)** Bursting pressure test.

The adhesion property of nerve repair materials directly determines their ability to achieve stable bridging of nerve breaks within the moist, dynamic physiological environment. Researchers have developed and widely applied a series of standardized testing methods to scientifically evaluate the adhesion property between biomaterials and nerve tissue (Figure 4).

Visual assessment is a straightforward and quick method (Figure 4A). Researchers directly attach biomaterials to various substrate surfaces, including planar tissue (such as the gastrocnemius muscle and liver) and curved tissue (such as the heart and spleen) (Deng et al., 2023; Ren et al., 2023). Subsequently, the adhesive properties of the material are visually demonstrated using methods like vertical lifting or peeling.

The adhesion strength at the material-substrate interface can also be evaluated through lap-shear test (Figure 4B). This method involves sandwiching the adhesive material between two substrates to form a lap shear joint, which is then subjected to tensile loading on a universal testing machine until separation occurs (Deng et al., 2023). The maximum load recorded during separation is divided by the bonded area to yield the adhesion strength (typically reported in kPa). This method intuitively simulates the scenario where materials endure shear stress in practical applications, serving as a core metric for evaluating their adhesion reliability (Lee et al., 2024; Wang et al., 2022).

Peeling test is another commonly used method for quantifying the adhesive energy between materials and biological tissues (Figure 4C). Based on the peeling angle, it is categorized into 180° peeling test (T-peeling test) and 90° peeling test (Freedman et al., 2022). First, the material is attached to the tissue (e.g., pig skin), and then one side is peeled away from the tissue surface at a constant rate. Finally, the peak average force is divided by the adhesion width to calculate the adhesion energy (typically reported in J/m^2^), reflecting the ability to resist peel failure (Zheng et al., 2025).

Additionally, tensile test provides another method for evaluating adhesion strength by determining the force required to separate the adhesive (such as hydrogel) from the tissue surface (Figure 4D), with adhesion strength reported in kPa (Deng et al., 2023). The evaluation method is similar to the lap-shear test (Zheng et al., 2025).

Moreover, the bursting pressure test has also been adopted by some researchers to evaluate the adhesion performance of materials (Figure 4E) (Shen et al., 2025). Adhesive materials are attached to the surface of perforated tissue or materials, and by filling a sealed container with liquid or gas to impose pressure, the adherence strength of the material is judged based on the maximum pressure value (often in mmHg) recorded in the container (Zheng et al., 2025).

Given the complex and dynamic microenvironment of PNI, it is crucial to evaluate adhesive performance of materials under different environmental conditions. Seong et al. incubated the bonded patch material with biological tissue under a range of humidity levels (20%, 50%, 80%), subsequently evaluating the durability of the adhesion (Seong et al., 2024). In the 180° peeling test conducted by Wang and colleagues, Zn^2+^ solutions of different concentrations were added to the material-tissue adhesion interface, to detect the alteration in attachment capability (Wang et al., 2022).

## 6 Functionalization of nerve repair adhesion interface

**FIGURE 5:**
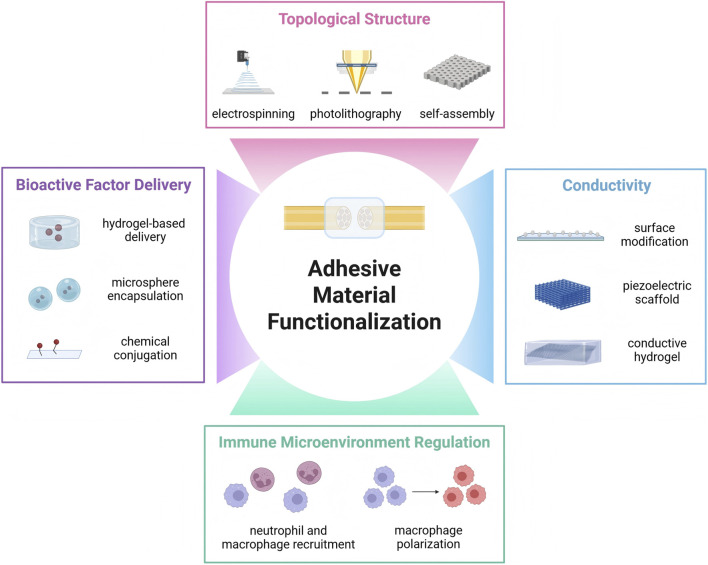
Functionalization of adhesive nerve repair materials. Besides robust adhesion performance, ideal nerve injury repair materials also have other indispensable requirement to achieve biological functions, such as bioactive factor delivery, conductivity, topological structure and immune microenvironment regulation.

Ideal peripheral nerve repair adhesive materials should not merely serve as mechanical bridging adhesives but function as active platforms that facilitate nerve regeneration. Current research focuses on mimicking the complex biological functions of natural nerves through multifunctional design to efficiently boost regeneration. This functionalization strategy aims to empower materials with active biological efficacy, including: delivering bioactive factors to provide chemical signaling guidance; incorporating conductivity to restore electrophysiological signal and provide electrical stimulation; constructing biomimetic topological structures to offer physical contact guidance for cells; and regulating the immune microenvironment to manage inflammatory responses (Figure 5).

### 6.1 Bioactive factor delivery

Superior regeneration of peripheral nerves requires the physical support of bridging defects, but also relies on the precise regulation of multiple neurotrophic factors. While simple adhesion interfaces can achieve stable and robust dissection alignment, integrating bioactive factors into adhesive materials to construct drug delivery systems represents a promising strategy for functional repair (Gu et al., 2014). Controlled release of signaling molecules enables intervention in the regeneration, promoting neuronal survival, guiding axonal directional extension, and stimulating Schwann cell proliferation and myelination (Escobar et al., 2022). Currently, bioactive factors commonly applied in nerve repair include nerve growth factor (NGF), brain-derived neurotrophic factor (BDNF), glial-derived neurotrophic factor (GDNF), vascular endothelial growth factor (VEGF), insulin-like growth factor 1 (IGF-1), fibroblast growth factor 2 (FGF2) and netrin-1. These factors may be administered individually or in combination to achieve rapid regeneration of injured nerves (Li et al., 2020). And they can be loaded and released through various methods, such as chemical conjugation, microsphere or nanoparticle encapsulation, and hydrogel-based delivery systems (Lin et al., 2024; Marcus et al., 2018). A study constructed a controlled-release nerve conduit by encapsulating FGF2 within hydrogel. This system sustained FGF2 release for over 30 days and demonstrated effective repair in long-segment nerve defect models (Rodriguez-Sanchez et al., 2025). Sophisticated material design endows the nerve repair material with potent interfacial adhesion capability while enabling the loading and release of bioactive factors within the injured micro-environment. This achieves synergistic material fixation and biochemical signaling, driving high-quality regeneration of peripheral nerve. Cai et al. developed a graphene-based neurotrophic conduit featuring robust adhesion and sustained *in vivo* delivery of netrin-1, significantly accelerating PNI repair and inhibiting muscle atrophy (Cai Y et al., 2022).

Extracellular matrix (ECM) constitutes the natural microenvironment of nerve. Its components include molecules such as collagen, hyaluronic acid, fibrin, and laminin, and it is also rich in various bioactive factors like NGF, IGF-1, and FGF (Li T et al., 2021). These components regulate the nerve repair process through multiple mechanisms: for example, fibronectin promotes Schwann cell spreading, while laminin enhances Schwann cell proliferation and migration (Xu et al., 2020). In recent years, researchers have developed biomimetic ECM materials based on silk fibroin and chitosan. These systems possess controllable degradation rates, suitable mechanical strength, and excellent biocompatibility, demonstrating remarkable effects in promoting nerve repair (Guan et al., 2023; Liu et al., 2024). Furthermore, the ECM serves as an excellent carrier for bioactive molecules, effectively delivering neurotrophic factors such as NGF, FGF, and neurotrophic vitamins (B1, B6, B12), thereby regulating the microenvironment and enhancing nerve regeneration (Li T et al., 2021; Viezuina et al., 2025). For example, a study developed an ECM-based conductive hydrogel bandage whose surface aldehyde groups form covalent bonds with amino groups in tissues, enabling stable adhesion to neural tissues. Meanwhile, the loaded bioactive factors within the hydrogel effectively modulate Schwann cell behavior and promote axon regeneration (Wang et al., 2023).

### 6.2 Conductivity

As electrically excitable tissues, the peripheral nervous system functions primarily through the generation and conduction of electrical signals. Therefore, equipping nerve repair materials with interfacial adhesion capabilities and electrical conductivity, beyond bridging damaged nerve ends, facilitates the restoration of physiological electrical signal continuity and regulates cellular behavior, thereby significantly boosting nerve regeneration effects (Ghosh et al., 2025; Guo and Ma, 2018; Wang et al., 2025).

Current conductive materials applied for neural repair primarily include: (1) conductive nanoparticles, such as gold nanoparticles, carbon-based nanomaterials, and graphene nanomaterials, which exhibit excellent biocompatibility and electrical properties (Rahman et al., 2023; Sharifi et al., 2023); (2) organic conductive polymers, including polyaniline and polypyrrole (PPy), with advantages such as simple synthesis, tunable structures, and high adaptability (Yao et al., 2021); (3) piezoelectric materials, encompassing piezoelectric polymers (e.g., PEDOT) and piezoelectric nanoparticles (e.g., barium titanate nanoparticles), can respond to mechanical stimuli and convert it into electrical signals (Candito et al., 2022; Yao et al., 2021). These materials can be utilized to construct functional nerve repair conduits via multiple strategies, including: (1) surface modification, such as coating conduits with conductive PPy; (2) matrix doping, like fabricating conductive scaffolds by compositing graphene with PCL; (3) constructing piezoelectric scaffolds capable of mechano-electrical conversion using piezoelectric materials like zinc oxide; (4) developing conductive hydrogels by incorporating materials such as graphene to build conductive networks (Kim et al., 2023; Wang et al., 2025).

Yan et al. developed a conductive adhesive hydrogel using cellulose nanotubes coated with PEDOT, which incorporates a uniformly deposited layer of PDA. The material exhibits excellent adhesion and conductivity, providing an optimal microenvironment for neuronal growth following traumatic brain injury and promoting the recovery of neural function (Yan et al., 2023). Jin and colleagues showed an adhesive conductive nerve bandage composed of hyaluronic acid hydrogel fibers crosslinked with boronic acid esters and PPy-coated hydrogel fibers, which respectively provide strong adhesion and conductivity to accelerate damaged nerve regeneration (Jin et al., 2025). Additionally, Wang et al. utilized extracellular matrix, oxidized polysaccharides, and PEDOT:PSS to prepare a conductive hydrogel. The aldehyde groups on hydrogel undergo Schiff base reactions with amino groups on tissues surface to achieve tissue adhesion, while PEDOT:PSS, as the conductive component, ensures the excellent electrical conductivity, thereby promoting axonal regeneration and functional recovery after nerve injury (Wang et al., 2023).

### 6.3 Topological structure

After PNI occurs, the injured nerve fiber firstly experiences Wallerian degeneration, and mature Schwann cells de-differentiate to form reparative cells and degrade the myelin sheath, creating a suitable microenvironment for nerve axon regeneration (Tricaud and Park, 2017). Later, Schwann cells proliferate to form Büngner bands, which act as scaffolds to guide nerve fiber regeneration and ultimately reinnervation of the target organ (Endo et al., 2022). Therefore, besides possessing strong interface adhesion, the ideal nerve repair material should be designed with specific physical topologies to guide Schwann cell migration, promote ordered axonal growth, and ultimately achieve functional recovery.

There are several approaches to constructing topological structures: electrospinning enables the fabrication of micro- and nano-scale fiber scaffolds through high-voltage electrostatic forces, allowing precise tuning of fiber orientation; phase separation induces polymer chains to rearrange and assemble, forming ordered structures by separating homogeneous solutions; photolithography, as a high-precision processing technique, can etch micrometer or nanometer-scale intricate patterns onto material surfaces; self-assembly utilizes intermolecular interactions to spontaneously construct ordered topological structures (Agrawal et al., 2021; Al-Hadeethi et al., 2023; Feng et al., 2025; Hu et al., 2022; Wu et al., 2023). Hu et al. coated the Morpho butterfly wing-inspired scaffold with reduced graphene oxide and BDNF-encapsulated GelMA hydrogel, imparting excellent conductivity and sustained BDNF release while preserving the original topological structure, ultimately demonstrating a promising peripheral nerve repair approach (Hu et al., 2022). Feng and colleagues prepared a topologically structured conductive hydrogel using polyvinyl alcohol and PEDOT:PSS, which exhibited excellent nerve growth promotion and sciatic nerve repair effects (Feng et al., 2025).

### 6.4 Immune microenvironment regulation

In PNI, the immune microenvironment is also a critical factor determining the speed and success of neural regeneration. During the demyelination phase, Schwann cells secrete chemokines to recruit various immune cells, predominantly M1 macrophages, to collaboratively clear myelin debris. However, these pro-inflammatory immune cells also release high levels of inflammatory cytokines such as TNF-α and IL-6, thereby triggering local inflammatory responses (Huang et al., 2022; Zigmond and Echevarria, 2019). Successful nerve repair depends on the polarization of macrophage from pro-inflammatory M1 phenotype to anti-inflammatory M2 phenotype. M2 macrophages secrete anti-inflammatory mediators like IL-10 and TGF-β, which effectively suppress inflammation, promote Schwann cell proliferation and migration, thereby accelerating axonal regeneration and functional recovery (Wang et al., 2023; Zigmond and Echevarria, 2019).

Therefore, ideal nerve repair materials should not only possess excellent interfacial adhesion properties but also actively regulate the immune microenvironment, with promoting macrophage polarization toward the M2 phenotype being a key strategy to achieve this function. Sun et al. fabricated lithium-magnesium-silicon bioceramics containing scaffolds that effectively recruit macrophages and facilitate their polarization, thereby promoting regeneration of damaged nerves (Sun et al., 2023). Wang and colleagues formulated a nerve bandage loaded with IPA, with its aldehyde group bonding to amino groups on tissue for adhesion. The released IPA facilitates recruited neutrophils to clear myelin debris and promote axonal growth (Wang et al., 2023). Xiao et al. developed a hydrogel with tissue adhesion through dopamine grafting, which also modulates the microenvironment by altering immune cell phenotypes, ultimately facilitating repair of injured nerves (Xiao et al., 2023).

## 7 Summary and perspectives

As a key element of nerve regeneration and functional recovery, adhesion interfaces of materials for PNI repair have witnessed significant research progress in recent years. This paper provides a systematic review of the up-to-date research progress in biomaterial adhesion interfaces for PNI repair. We first demonstrate the critical importance of robust and stable adhesion interfaces for constructing effective microenvironments for nerve regeneration. Subsequently, the article addresses multiple mechanisms enabling material-tissue adhesion (including mechanical interlocking, electrostatic bonding, molecular bonding, and permeation), and presents a categorized view of adhesion interfaces based on material source (natural and synthetic polymers), as well as discusses dry adhesion and wet adhesion. Additionally, we summarize current common methods for evaluating adhesion properties, and highlight functionalization strategies for adhesion interface, such as delivery of bioactive factors, imparting conductivity, topological structure design, and immune microenvironment regulation. These functionalization approaches significantly contribute to the efficiency of complex nerve repair by adhesive materials.

Despite remarkable achievements in this field, future research still faces numerous challenges and opportunities in pursuit of the ultimate goal of clinical translation. The following directions warrant further exploration: (1) promote the interface design of multi-mechanism synergy, realize the synergistic coupling of mechanical interlocking and covalent binding, electrostatic attraction and hydrogen bonding through the precise regulation of molecular structure, to meet the dynamic needs of nerve regeneration; (2) develop dynamically responsive adhesion interfaces, use pH-sensitive, temperature-sensitive and other materials to construct adhesion systems with regulatory capabilities, to achieve the precise regulation of adhesion strength and bioactivity during the repair process (Gao et al., 2025); (3) utilize additive manufacturing (e.g., 3D printing) to create personalized repair materials tailored to specific nerve injuries, which allows precise control over the strength and bioactivity, ultimately enhancing the effect of nerve repair (Rizwana et al., 2024; Rizwana, et al., 2025); (4) achieve multifunctional material design by integrating bioactive factors delivery, conductivity, topological structure, immune regulation, and strong adhesion capability within a single material system, coupled with temporal and spatial coordination of these functions; (5) establish a robust clinical translation and evaluation system, develop animal models that more closely mimic human pathophysiological states, alongside standardized and normalized assessment methods, thus advancing outstanding research outcomes into clinical applications.

In summary, future research should continue to advance the basic exploration and clinical translation of the adhesion interface of nerve repair materials. Through the profound integration of materials science, biology, medicine, and engineering, the efficient regeneration and repair of damaged nerves can be achieved, providing promising solutions for clinical treatment.
